# Late pandemic COVID-19 positivity at the time of thrombectomy is associated with poor outcomes and tandem carotid pathology

**DOI:** 10.3389/fneur.2025.1513124

**Published:** 2025-02-24

**Authors:** Lewis John Rubin Thompson, Clifton Houk, Nathaniel R. Ellens, Gurkirat Singh Kohli, Derrek Schartz, Diana Proper, Tarun Bhalla, Matthew T. Bender, Thomas K. Mattingly

**Affiliations:** ^1^Department of Neurosurgery, University of Rochester Medical Center, Rochester, NY, United States; ^2^Department of Imaging Sciences, University of Rochester Medical Center, Rochester, NY, United States

**Keywords:** ischemic stroke, COVID-19, carotid, endovascular, mechanical thrombectomy

## Abstract

**Objectives:**

COVID-19 is an independent risk factor for ischemic stroke. Studies from early in the pandemic show increased rates of unfavorable recanalization, poor outcomes, and mortality in patients who were COVID-19 positive at the time of mechanical thrombectomy. However, there are currently no studies examining these parameters during the later pandemic when circulating variants were less virulent.

**Materials and methods:**

We performed a retrospective review of mechanical thrombectomies from 12/2020 to 3/2023. Patients who were COVID-19 positive at the time of thrombectomy were included. Demographic, procedural, and 90-day functional outcomes were evaluated.

**Results:**

Of 306 patients undergoing mechanical thrombectomy for acute ischemic stroke between 12/2020 and 3/2023, 18 were COVID-19 positive. Compared with the COVID-19 negative cohort, there were lower rates of favorable recanalization (73% vs. 92%, *p* = 0.03) and good functional outcomes (26% vs. 49%, *p* = 0.06), but greater tandem carotid pathology (42% vs. 12%, *p* < 0.01), and a higher mortality rate (53% vs. 26%, *p* = 0.02). However, COVID-19 positive status did not predict outcomes in multivariable analysis when controlled for age, NIHSS, IV tPA, recanalization status, and tandem carotid pathology.

**Conclusion:**

Late in the pandemic, outcomes remained comparable to those observed in the early pandemic for patients positive for COVID-19 at the time of mechanical thrombectomy. This case series also demonstrates increased tandem carotid pathology in the COVID-19 cohort. While COVID-19 may not influence outcome to the degree that age and NIHSS do, the excess mortality continues to suggest a negative effect despite lower virulence.

## Introduction

Emerging evidence continues to illustrate the multi-system impact of SARS-CoV-2 infection, including disruption of the endoluminal space. SARS-CoV-2 is believed to cause endothelial disfunction and induce a hypercoagulable state due to the presence of angiotensin converting enzyme (ACE)-2 on the surface of vascular endothelial cells and dysregulation of the immune response (1–4). These mechanisms are believed to underly the increased rate of thrombotic events, including acute ischemic stroke (AIS), observed in COVID-19 patients (5, 6).

In single-center and multicenter studies examining outcomes following mechanical thrombectomy in COVID-19 positive patients with AIS, there have been conflicting results. Many studies indicate a significantly higher mortality rate for patients who test positive for COVID-19 at the time of mechanical thrombectomy, while some have demonstrated equivalent outcomes between patient groups (7–13). However, essentially all data are from 2020, early in the pandemic. As the pandemic progressed, a series of COVID-19 variants were seen, with increased transmissibility but decreased virulence (14–16). Here we present a single-center retrospective analysis of patients who tested positive for COVID-19 at the time of mechanical thrombectomy from 2021 to 2023 to evaluate outcomes in the later stages of the pandemic.

## Materials and methods

This is a single center retrospective review of mechanical thrombectomies performed from 12/1/2020 to 3/23/23. Using an institutional database, we evaluated demographic, procedural and 90 day functional outcome data in setting of COVID-19 status. We had routine in-house PCR-based COVID-19 testing established during the period studied, such that COVID-19 status could be established at or just after the time of thrombectomy. Given that COVID-recovered patients often still test positive, and the established increased perioperative risk, we included both COVID-19 positive and recently COVID-19 recovered in the COVID-19 positive cohort. IV tPA was administered to patients according to practice guidelines out to 4.5 h. Case fatality rates were determined using data from Monroe County available through the New York state Department of Health.

We evaluated categorical variables with Fisher’s exact or Chi-squared tests and continuous variables with Welch’s *T* test. Univariable analysis of 90-day functional outcome was performed for candidate variables. Variables with a *p*-value of <0.2 were included in the multivariable logistic regression analysis for favorable 90-day functional outcomes (defined as mRS 0–2) and mortality, adjusted for age, National Institute of Health Stroke Scale (NIHSS), COVID-19 status, and procedural variables. Statistical significance was set at *p* < 0.05. Analysis was performed with PRISM 10.1.1 (Graphpad, San Diego, CA) and R studio (2024.12.0.467, R version 4.4.1).

## Results

A total of 306 mechanical thrombectomies were performed during the time frame of December 1, 2020 to March 23, 2023. Of these we identified 18 patients who were COVID-19 positive with 19 thrombectomies (Table 1). The only patient considered COVID-19 recovered had a second large vessel occlusion (LVO) on the contralateral side on post-operative day one. One third of patients received intravenous tissue plasminogen activator (IV tPA). All patients had anterior circulation LVO. Tandem cervical internal carotid artery (ICA) pathology was frequent (8/19 cases, 42%) including 2 carotid occlusions, 2 non-flow limiting stenoses (<70%), 2 flow-limiting stenoses (>70%), 1 dissection and 1 non-occlusive thrombus with <50% stenosis. By contrast, the COVID-19 negative population had a 12% incidence of cervical carotid pathology (*p* < 0.01) (Table 2). In patients with carotid pathology causing flow limiting stenosis (>70%) or occlusion, there was no difference between COVID Positive and Negative groups. The case fatality rate for Monroe County from the early pandemic (March 2020–November 2020) was 2.2 and 0.95% in the late pandemic (December 2020–March 2023).

**Table 1:** COVID-19 cohort characteristics and demographics.

COVID-19 Positive at arrival?	Date arrival	NIHSS	IV tPA	Clot location	Cervical ICA pathology	final TICI score	Additional procedures	LOS	Discharge disposition	90d mRS	Cause of CVA (TOAST)	Symptomatic COVID-19?	D dimer	Notes
Yes	12/20	28	Yes	L M1, M3	0	3		8	Deceased	6	2	Yes		
Yes	1/21	16	Yes	R ICAT	0	2b		6	Deceased	6	4	Yes	2.19	
Yes	1/21	16	Yes	R M1	0	2a		9	Home	6	5	No		Seizure/death at home
Recovered	3/21	24	No	L ICAT	0	3	R M1 POD1	10	See below	See below	2	No		Same patient
Recovered	3/21	NR	No	R M1	0	3		10	Deceased	6	4	No	2.98	Same patient
Yes	3/21	9	Yes	R ICA, M1	Reoccluded	2b		6	Rehabilitation	3	1	No		
Yes	7/21	14	No	R M1	0	3		11	SNF	4	2	No		
Yes	9/21	23	No	L M1	0	3		0	Deceased	6	4	No		
Yes	11/21	13	No	L M2	0	2a		9	Deceased	6	2	No		
Yes	12/21	9	No	L M2	Atherosclerosis	2a		5	Home	1	2	No		
Yes	12/21	7	No	L M1	Sub-occlusive thrombus	2b		18	SNF	2	4	No		
Yes	4/22	23	No	L ICA	Occlusion	3		24	Rehabilitation	5	2	No		
Yes	5/22	31	No	R M1	Stenosis >70%	2b	R CEA POD 21; L CEA POD 32	46	Deceased	6	1	No		Multifocal CVA post CEA
Yes	5/22	7	No	R ICA	Stenosis >70%	3	CAS	14	Home	1	1	No		
Yes	5/22	8	No	L M1	0	2b		3	Home	1	2	No		
Yes	8/22	5	Yes	R M3	0	2b		76	SNF	5	4	No		
Yes	8/22	15	No	R ICAT	0	3		2	Home	0	2	No		
Yes	2/23	11	No	R ICAT	Dissection	2a	Acute CAS	33	Deceased	6	1	No		PE
Yes	3/23	13	Yes	R M1	Atherosclerosis	0		13	Deceased	6	1	No		

CAS, carotid artery stenting; CEA, carotid endarterectomy; CVA, cerebrovascular accident; ICA, internal carotid artery; ICAT, internal carotid artery terminus; LOS, length of stay; mRS, modified Rankin score; NIHSS, National Institute of Health Stroke Scale; PE, pulmonary embolism; POD, post-operative day; SNF, skilled nursing facility; TICI, thrombolysis in cerebral infarction; TOAST, Trial of Org 10,172 in Acute Stroke Treatment; tPA, tissue plasminogen activator.

**Table 2:** Study cohort characteristics.

	COVID-19 positive (*n* = 19)	COVID-19 negative (*n* = 287)	*p*-value
Age (mean +/− SD)	71.6 +/− 11.3	70.2 +/−14.6	0.62
NIHSS (mean +/− SD)	15.1 +/−7.7	15.0 +/−7.0	0.94
TICI 2b-3 (%)	14 (73)	263 (92)	**0.03**
90d mRS 0–2 (%)	5 (26)	140 (49)	0.06
90d mortality (%)	10 (53)	72 (26)	**0.02**
Cervical ICA pathology (%)	8 (42)	34 (12)	**<0.01**
IV tPA given?	6 (33)	88 (31)	0.97
LKW to groin time, minutes, mean +/− SD	381.1 +/− 264.3	403.2 +/− 323.6	0.73
LKW to reperfusion time, minutes, mean +/− SD	435.1 +/− 279.1	438.8 +/− 316.1	0.95
Groin to reperfusion time, minutes, mean +/− SD	49.7 +/− 31.3	44.1 +/− 24.0	0.47

ICA, internal carotid artery; LKW, last known well; mRS, modified Rankin score; NIHSS, National Institute of Health Stroke Scale; SD, standard deviation; TICI, thrombolysis in cerebral infarction. Bolded *p*-values indicate those that are statistically significant.

**Figure 1:**
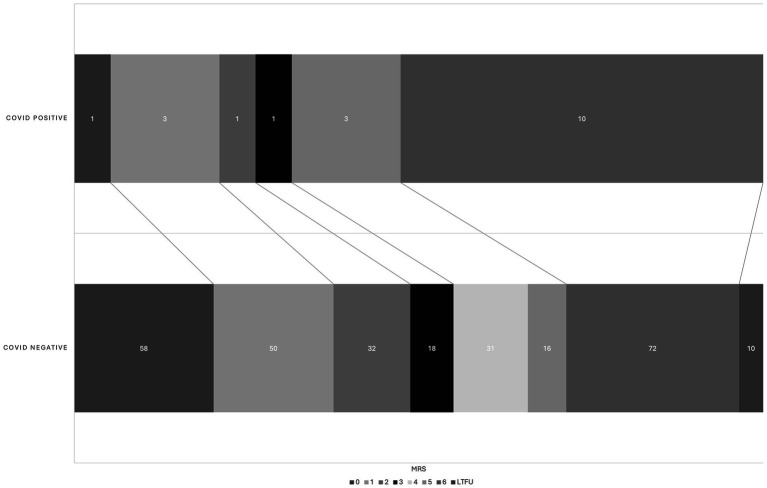
Graphical shift of 90d mRS in COVID positive and COVID negative cohort. There is a significant between-group difference by Mann–Whitney *U* test (*p* < 0.01). LTFU, lost to follow up.

There was no difference in NIHSS between the COVID-19 positive and COVID-19 negative groups (mean 15.1 +/− 7.7 vs. 15.0 +/− 7.0, *p* = 0.94). Good procedural outcomes (TICI 2b-3) were achieved in 14/19 cases (73%), which was less than the 92% achieved in the COVID negative population (*p* = 0.03). There were no statistically significant differences in procedural metrics such as last know well (LKW) to groin time, LKW to reperfusion time, and groin to reperfusion times. Good functional outcomes (90-day mRS 0–2) were achieved in 5/19 COVID positive cases (26%), while 140/297 (49%) achieved this in the COVID-19 negative cohort (*p* = 0.06) (Table 2). However, the 90-day mortality was significantly higher in the COVID positive cohort (10/19, 53%), compared with COVID-19 negative group (*p* = 0.02) (Figure 1). Likewise, there were significant differences in between-group outcomes (Mann–Whitney *U* test; *p* < 0.01). In univariate analysis, COVID positive status was significantly associated with poor outcomes (OR 0.3571, CI 0.1154–0.9334, *p* = 0.048) and mortality (OR 3.322, CI 1.251–8.426, *p* = 0.015) (Tables 3, 4). We then analyzed the effect of COVID-19 positivity on 90-day functional outcomes in a multivariable logistic regression model controlled for age, NIHSS, recanalization score, IV tPA administration, and presence of tandem pathology (Table 3). This demonstrated that younger age (OR 0.97, CI 0.95–0.99, *p* = <0.01), lower NIHSS (OR 0.88, CI 0.84–0.91, *p* = <0.01), and administration of IV tPA (OR 2.03, CI 1.13–3.70, *p* = 0.02) were significant predictors of good outcome, whereas COVID-19 positive status was not a predictor of good outcome (OR 0.44, CI 0.122–1.395, *p* = 0.18) (Table 3). Logistic regression modeling using mortality as the endpoint again showed COVID positivity was a significant predictor in univariate analysis (OR 3.22, CI 1.25–1.84, *p* = 0.015) but dropped out in a multivariable model (OR 2.61, CI 0.84–8.15, *p* = 0.096) controlled for age, NIHSS, IV tPA, tandem carotid pathology, and recanalization grade (Table 4).

**Table 3:** Univariable and multivariable logistic regression of factors impacting good outcome (mRS 0–2).

Univariable			
Variable	OR	95% CI	*P*-value
COVID+	0.357	0.115 to 0.933	**0.048**
Patient age	0.998	0.995 to 1.001	0.189
Initial NIHSS	0.978	0.9638 to 0.992	**0.002**
IV tPA	0.827	0.626 to 1.091	0.181
Cervical ICA pathology	0.640	0.335 to 1.188	0.163
TICI 2b-3	0.588	0.260 to 1.264	0.183

Multivariable			
Variable	OR	95% CI	*P*-value
COVID+	0.4412	0.1218 to 1.395	0.180
Patient Age	0.9680	0.9486 to 0.9868	**<0.001**
Initial NIHSS	0.8754	0.8367 to 0.9128	**<0.001**
IV tPA	2.0270	1.129 to 3.698	**0.020**
Cervical ICA pathology	0.4919	0.2126 to 1.104	0.090
TICI 2b-3	0.6608	0.2494 to 1.683	0.390

ICA, internal carotid artery; mRS, modified Rankin score; NIHSS, National Institute of Health Stroke Scale; OR, odds ratio; TICI, thrombolysis in cerebral infarction; tPA, tissue plasminogen activator. Bolded *p*-values indicate those that are statistically significant.

**Table 4:** Univariable and multivariable logistic regression of factors impacting 90-day mortality (mRS 6).

Univariable			
Variable	OR	CI	*p*-value
COVID+	3.224	1.251 to 8.426	**0.015**
Patient age	1.039	1.019 to 1.062	**<0.001**
Initial NIHSS	1.132	1.085 to 1.184	**<0.001**
IV tPA	0.981	0.558 to 1.695	0.945
Cervical ICA pathology	1.277	0.609 to 2.564	0.502
TICI 2b-3	0.431	0.193 to 0.983	**0.041**

Multivariable			
Variable	OR	CI	*p*-value
COVID+	2.605	0.835 to 8.149	0.096
Patient age	1.036	1.013 to 1.062	**0.003**
Initial NIHSS	1.144	1.093 to 1.203	**<0.001**
IV tPA	0.834	0.430 to 1.582	0.583
Cervical ICA pathology	1.31	0.526 to 3.121	0.549
TICI 2b-3	0.302	0.114 to 0.792	**0.015**

Bolded *p*-values indicate those that are statistically significant.

## Discussion

### Elevated mortality rate even in the late pandemic

This single-center, retrospective study found a higher mortality rate (53%) in patients who tested positive for COVID-19 at the time of mechanical thrombectomy than in patients who tested negative. COVID-19 positive patients also had a lower rate of favorable revascularization. Multivariable logistic regression analysis showed that COVID-19 positive status at the time of thrombectomy was not associated with favorable 90-day functional outcomes. The data reported in this study are concordant with previous studies that demonstrated lower rates of favorable recanalization and worse outcomes in patients with COVID-19 at the time of mechanical thrombectomy (7–11). However, previous literature is focused on the early pandemic (3/2020–12/2020), while our case series is exclusively in the late pandemic.

### Late pandemic COVID-19 virulence is lower

This study examined outcomes in the later stages of the COVID-19 pandemic, during which dominant circulating strains of SARS-CoV-2 were shown to have lower virulence (14–16). Nevertheless, our single center series shows patterns of lower recanalization, excess mortality, and a trend toward worse outcomes similar to that seen in the early pandemic. This has implications for additional waves of COVID-19, as SARS-CoV-2 has now established itself as an endemic virus; the virulence may have decreased, and those who come in COVID-19 positive may not be “symptomatic” in a traditional sense, yet they appear to continue to have higher risk of cerebrovascular events. In our series, only the two earliest patients were symptomatic. The decrease in virulence and overall mortality of SARS-CoV-2 infection is further suggested by the decrease in case fatality rates observed in our county from the early pandemic (March 2020 – December 2020) and the late pandemic (January 2021 and beyond). An open question is whether vaccination has a positive impact on this cerebrovascular event risk. In a recent review of COVID and cardiovascular events, it appears that vaccination does indeed reduce overall CVE risk when compared with unvaccinated adults (17).

### Immuno-thrombosis in COVID-19

We assessed the suspected cause of the stroke based on established TOAST criteria (18). The majority of vessel occlusions in COVID-19 positive patients were either cardioembolic in origin or attributed to a presumed hypercoagulable state (TOAST 4). COVID-19 has been well documented as an independent risk factor for AIS (5, 6, 19). The underlying mechanisms of thrombotic events attributable to SARS-CoV-2 infection are thought to be endothelial disruption and dysregulation of the immune response, leading to hypercoagulability and hyperinflammation. Endothelial disruption is believed to occur due to direct infection of endothelial cells by SARS-CoV-2 via binding to ACE-2, leading to cellular damage and apoptosis, thus disrupting the antithrombotic activity of intact epithelium (3, 20, 21). Although classically associated with the pulmonary epithelium, ACE-2 receptors are widely distributed throughout the vascular endothelium (22). COVID-19 is also associated with high levels of proinflammatory cytokines such as interleukin-6 (IL-6), IL-1B, IL-18, and granulocyte-macrophage colony-stimulating factor, a state commonly referred to as “cytokine storm” (23, 24). This hyperinflammatory state can cause intravascular release of neutrophil extracellular traps (NETs), immune complexes which are capable of activating platelet aggregation and the extrinsic and intrinsic pathways of the coagulation cascade (3). NETs are believed to be a conserved mechanism of the innate immune system capable of limiting the circulation of pathogens through the microcirculation (25, 26). However, in a state of widespread endothelial inflammation, this mechanism can lead to significant disruption of the microcirculation and is likely a key factor contributing to both the lower rate of optimal recanalization and poor outcomes observed in COVID-19 patients who experience large-vessel AIS.

### Enriched carotid disease

COVID-19 positive patients in this study also had higher rates of concomitant cervical internal carotid artery (ICA) pathology including atherosclerosis, stenosis, sub-occlusive thrombosis, and one case of dissection. There have been some reports in the literature of an association between COVID-19 and atherothrombosis of the ICA (27–30). Esenwa et al. (29) described a series of three COVID-19 patients with known mild carotid atherosclerosis who developed AIS due to carotid thrombosis. They postulated that endotheliitis induced by SARS-CoV-2 may destabilize otherwise quiescent atherosclerotic plaques and cause thrombosis (29). Gulko et al. (28) described two patients who were COVID-19 positive at the time of acute thrombosis of the carotid bifurcation with concomitant evidence of acute and subacute infarcts of the cerebral vasculature; in both cases, there was no evidence of intra- or extracranial atherosclerotic disease in their patient (28). Viguier et al. (30) described a 73-year-old with no vascular risk factors who developed fulminant common carotid thrombosis 1 week after developing respiratory symptoms related to COVID-19; imaging ultimately revealed only a thin, non-ulcerating plaque in the common carotid artery (30). An international, multicenter study conducted by Dmytriw et al. (10) also demonstrated that COVID-19 positive patients with LVO were more likely to have stroke due to large vessel atherosclerosis, where as non-COVID-19 LVO was more likely to be of cardioembolic origin. While these cases and studies suggest an elevated risk of carotid pathology in COVID-19 patients, our study is the first that shows a statistically significant association between carotid disease and COVID-19 patients with AIS.

### Limitations

The primary limitations of this study are its retrospective design and lack of randomization. As a single-center study, our sample size was relatively modest, which may limit the generalizability of our findings to broader populations. Additionally, due to inconsistent documentation in the early pandemic, we did not have COVID-19 test data for patients who underwent mechanical thrombectomy between March 2020 and December 2020 thus reducing the internal validity of this study.

Assessing the suspected cause of the stroke is difficult. While we assessed that a quarter of the COVID-19 positive cohort had a hypercoagulable mechanism, only two of the patients had any objective measure such as D-dimer. There is often overlap in mechanisms. The patient with a carotid artery dissection who underwent a carotid artery stent ultimately passed from a suspected pulmonary embolism. A few patients’ etiologies were clear, such as the patient with bilateral embolic events a day apart, who was found to have an elevated D-dimer. However, this patient was also deemed COVID-recovered, as it is common for patients to test positive for weeks after their symptoms have resolved.

## Conclusion

In the later stages of the COVID-19 pandemic, mortality rates in COVID-19 positive patients undergoing mechanical thrombectomy remain high. Despite the lower virulence of later circulating variants, SARS-CoV-2 infection remains a neurovascular concern as demonstrated by a higher incidence of tandem carotid pathology observed in this cohort. Further research is required to clarify if progressively less virulent strains of COVID-19 continue to influence stroke outcomes as the virus continues to evolve.

## Data Availability

The original contributions presented in the study are included in the article/supplementary material, further inquiries can be directed to the corresponding author.

## References

[1] Otifi HM Adiga BK. Endothelial dysfunction in Covid-19 infection. Am J Med Sci. (2022) 363:281–7. doi: 10.1016/j.amjms.2021.12.010, PMID: 35093394 PMC8802031

[2] Varga Z Flammer AJ Steiger P Haberecker M Andermatt R Zinkernagel AS. Endothelial cell infection and endotheliitis in COVID-19. Lancet. (2020) 395:1417–8. doi: 10.1016/S0140-6736(20)30937-5, PMID: 32325026 PMC7172722

[3] Bonaventura A Vecchié A Dagna L Martinod K Dixon DL van B. Endothelial dysfunction and immunothrombosis as key pathogenic mechanisms in COVID-19. Nat Rev Immunol. (2021) 21:319–29. doi: 10.1038/s41577-021-00536-9, PMID: 33824483 PMC8023349

[4] Kuba K Imai Y Rao S Gao H Guo F Guan B. A crucial role of angiotensin converting enzyme 2 (ACE2) in SARS coronavirus-induced lung injury. Nat Med. (2005) 11:875–9. doi: 10.1038/nm1267, PMID: 16007097 PMC7095783

[5] Katsoularis I Fonseca-Rodriguez O Farrington P Lindmark K Fors Connolly AM. Risk of acute myocardial infarction and ischaemic stroke following COVID-19 in Sweden: a self-controlled case series and matched cohort study. Lancet. (2021) 398:599–607. doi: 10.1016/S0140-6736(21)00896-5, PMID: 34332652 PMC8321431

[6] Belani P Schefflein J Kihira S Rigney B Delman BN Mahmoudi K. COVID-19 is an independent risk factor for acute ischemic stroke. AJNR Am J Neuroradiol. (2020) 41:1361–4. doi: 10.3174/ajnr.A6650, PMID: 32586968 PMC7658882

[7] Jabbour P Dmytriw AA Sweid A Piotin M Bekelis K Sourour N. Characteristics of a COVID-19 cohort with large vessel occlusion: a multicenter international study. Neurosurgery. (2022) 90:725–33. doi: 10.1227/neu.0000000000001902, PMID: 35238817 PMC9514728

[8] Styczen H Maus V Goertz L Köhrmann M Kleinschnitz C Fischer S. Mechanical thrombectomy for acute ischemic stroke in COVID-19 patients: multicenter experience in 111 cases. J Neurointerv Surg. (2022) 14:858–62. doi: 10.1136/neurintsurg-2022-018723, PMID: 35292572

[9] Zureigat H Alhusban M Cobia M. Mechanical Thrombectomy outcomes in COVID-19 patients with acute ischemic stroke: a narrative review. Neurologist. (2021) 26:261–7. doi: 10.1097/NRL.0000000000000360, PMID: 34734904 PMC8575107

[10] Dmytriw AA Ghozy S Sweid A Piotin M Bekelis K Sourour N. International controlled study of revascularization and outcomes following COVID-positive mechanical thrombectomy. Eur J Neurol. (2022) 29:3273–87. doi: 10.1111/ene.15493, PMID: 35818781 PMC9349405

[11] Qureshi AI Baskett WI Huang W Shyu D Myers D Raju M. Acute ischemic stroke and COVID-19: an analysis of 27 676 patients. Stroke. (2021) 52:905–12. doi: 10.1161/STROKEAHA.120.031786, PMID: 33535779 PMC7903982

[12] El-Qushayri AE Reda A Dahy A Azzam AY Ghozy S. The impact of COVID 19 on the outcomes of thrombectomy in stroke patients: a systematic review and meta-analysis. Rev Med Virol. (2023) 33:e2379. doi: 10.1002/rmv.2379, PMID: 35833712 PMC9349746

[13] Sawczyńska K Wrona P Kęsek T Wnuk M Chrzan R Homa T. Mechanical thrombectomy in COVID-19-associated ischaemic stroke: patient characteristics and outcomes in a single-Centre study. Neurol Neurochir Pol. (2022) 56:163–70. doi: 10.5603/PJNNS.a2022.0026, PMID: 35315928

[14] Pitsillou E Yu Y Beh RC Liang JJ Hung A Karagiannis TC. Chronicling the 3-year evolution of the COVID-19 pandemic: analysis of disease management, characteristics of major variants, and impacts on pathogenicity. Clin Exp Med. (2023) 23:3277–98. doi: 10.1007/s10238-023-01168-0 37615803

[15] Balint G Voros-Horvath B Szechenyi A. Omicron: increased transmissibility and decreased pathogenicity. Signal Transduct Target Ther. (2022) 7:151. doi: 10.1038/s41392-022-01009-8, PMID: 35525870 PMC9077027

[16] Suzuki R Yamasoba D Kimura I Wang L Kishimoto M Ito J. Attenuated fusogenicity and pathogenicity of SARS-CoV-2 omicron variant. Nature. (2022) 603:700–5. doi: 10.1038/s41586-022-04462-1, PMID: 35104835 PMC8942852

[17] Fundora MP Kamidani S Oster ME. COVID vaccination as a strategy for cardiovascular disease prevention. Curr Cardiol Rep. (2023) 25:1327–35. doi: 10.1007/s11886-023-01950-2 37688764

[18] Adams HP Jr Bendixen BH Kappelle LJ Biller J Love BB Gordon DL. Classification of subtype of acute ischemic stroke. Definitions for use in a multicenter clinical trial. TOAST. Trial of org 10172 in acute stroke treatment. Stroke. (1993) 24:35–41. doi: 10.1161/01.str.24.1.35, PMID: 7678184

[19] Kihira S Schefflein J Mahmoudi K Rigney B Delman BN Mocco J. Association of Coronavirus Disease (COVID-19) with large vessel occlusion strokes: a case-control study. AJR Am J Roentgenol. (2021) 216:150–6. doi: 10.2214/AJR.20.23847 32755225

[20] Wichmann D. Autopsy findings and venous thromboembolism in patients with COVID-19. Ann Intern Med. (2020) 173:1030. doi: 10.7326/L20-1206, PMID: 33316197

[21] Teuwen LA Geldhof V Pasut A Carmeliet P. COVID-19: the vasculature unleashed. Nat Rev Immunol. (2020) 20:389–91. doi: 10.1038/s41577-020-0343-0, PMID: 32439870 PMC7240244

[22] Clerkin KJ Fried JA Raikhelkar J Sayer G Griffin JM Masoumi A. COVID-19 and cardiovascular disease. Circulation. (2020) 141:1648–55. doi: 10.1161/CIRCULATIONAHA.120.046941, PMID: 32200663

[23] Fajgenbaum DC June CH. Cytokine Storm. N Engl J Med. (2020) 383:2255–73. doi: 10.1056/NEJMra2026131, PMID: 33264547 PMC7727315

[24] Mehta Y Dixit SB Zirpe KG Ansari AS. Cytokine storm in novel coronavirus disease (COVID-19): expert management considerations. Indian J Crit Care Med. (2020) 24:429–34. doi: 10.5005/jp-journals-10071-23415, PMID: 32863636 PMC7435090

[25] Engelmann B Massberg S. Thrombosis as an intravascular effector of innate immunity. Nat Rev Immunol. (2013) 13:34–45. doi: 10.1038/nri3345, PMID: 23222502

[26] Hickey MJ Kubes P. Intravascular immunity: the host-pathogen encounter in blood vessels. Nat Rev Immunol. (2009) 9:364–75. doi: 10.1038/nri2532, PMID: 19390567

[27] Casana R Domanin M Romagnoli S Bissacco D Malloggi C Grassi V. COVID-19 and supra-aortic trunks disease: review of literature about critical phase and sequelae. J Cardiovasc Surg. (2021) 62:535–41. doi: 10.23736/S0021-9509.21.12021-X, PMID: 34581553

[28] Gulko E Gomes W Ali S Al-Mufti F Mehta H. Acute common carotid artery bifurcation Thrombus: an emerging pattern of acute strokes in patients with COVID-19? AJNR Am J Neuroradiol. (2020) 41:E65–6. doi: 10.3174/ajnr.A6657, PMID: 32616583 PMC7658886

[29] Esenwa C Cheng NT Lipsitz E Hsu K Zampolin R Gersten A. COVID-19-associated carotid Atherothrombosis and stroke. AJNR Am J Neuroradiol. (2020) 41:1993–5. doi: 10.3174/ajnr.A6752, PMID: 32819896 PMC7658853

[30] Viguier A Delamarre L Duplantier J Olivot JM Bonneville F. Acute ischemic stroke complicating common carotid artery thrombosis during a severe COVID-19 infection. J Neuroradiol. (2020) 47:393–4. doi: 10.1016/j.neurad.2020.04.003, PMID: 32389423 PMC7196531

